# Challenges of Using Expansion Microscopy for Super‐resolved Imaging of Cellular Organelles

**DOI:** 10.1002/cbic.202000571

**Published:** 2020-11-11

**Authors:** Maximilian Büttner, Christoffer B. Lagerholm, Dominic Waithe, Silvia Galiani, Wolfgang Schliebs, Ralf Erdmann, Christian Eggeling, Katharina Reglinski

**Affiliations:** ^1^ MRC Human Immunology Unit MRC Weatherall Institute of Molecular Medicine University of Oxford Headley Way Oxford OX3 9DS UK; ^2^ Wolfson Imaging Centre MRC Weatherall Institute of Molecular Medicine University of Oxford Headley Way Oxford OX3 9DS UK; ^3^ Institute of Biochemistry and Pathobiochemistry Systemic Biochemistry Ruhr-University Bochum Universitätsstraße 150 44801 Bochum Germany; ^4^ Leibniz-Institute of Photonic Technologies & Institute of Applied Optic and Biophysics Friedrich-Schiller University Jena Max-Wien-Platz 1 07743 Jena Germany; ^5^ University Hospital Jena Bachstraße 18 07743 Jena Germany; ^6^ Institute for Anatomy and Cell Biology Martin-Luther-University Halle-Wittenberg Große Steinstraße 52 06108 Halle Germany

**Keywords:** bioorganic chemistry, cell organelles, expansion microscopy, peptides, STED microscopy

## Abstract

Expansion microscopy (ExM) has been successfully used to improve the spatial resolution when imaging tissues by optical microscopy. In ExM, proteins of a fixed sample are crosslinked to a swellable acrylamide gel, which expands when incubated in water. Therefore, ExM allows enlarged subcellular structures to be resolved that would otherwise be hidden to standard confocal microscopy. Herein, we aim to validate ExM for the study of peroxisomes, mitochondria, nuclei and the plasma membrane. Upon comparison of the expansion factors of these cellular compartments in HEK293 cells within the same gel, we found significant differences, of a factor of above 2, in expansion factors. For peroxisomes, the expansion factor differed even between peroxisomal membrane and matrix marker; this underlines the need for a thorough validation of expansion factors of this powerful technique. We further give an overview of possible quantification methods for the determination of expansion factors of intracellular organelles, and we highlight some potentials and challenges.

## Graphical abstract

**In Expansion Microscopy (ExM)** subcellular structures are imaged in isotropically expanded fixed samples, consequently allowing to resolve enlarged subcellular structures, otherwise hidden to standard microscopy. Upon comparison of the expansion factors of different cellular compartments in cells within the same gel, we found significant differences in expansion factors of a factor of above 2.

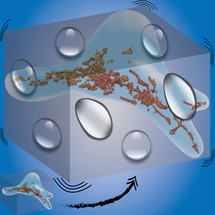

## Introduction

Expansion microscopy (ExM) was shown to be a good tool to increase the resolution in imaging biological samples by embedding them into a swellable acrylamide gel.[^1^, ^2^] The sample is fixed, permeabilized and crosslinked to the gel, which, when incubated in water, expands isotropically.

ExM has also been successfully combined with super‐resolution techniques such as single‐molecule switching based technique (STORM).^[3]^ and stimulated emission depletion (STED) microscopy. The latter combination of which has been employed to study microtubules,[^4^, ^5^, ^6^] cilia and centrioles.^[7]^ The combination of ExM with these techniques provides a promising tool to disclose details beyond the resolution limit of a super‐resolution microscope. Specifically, the crowded environment of intracellular organelles seems an interesting target for such an approach, as the isotropic enlargement of the sample can lead to a better separation of such signals. Here, it is of special interest to also define the localisation of potentially interacting proteins in respect to one another through analysing their colocalization.

We previously studied the colocalization of peroxisomal membrane proteins with STED microscopy^[8]^ and found these proteins to be distributed differently on different peroxisomes. A higher level of detail, than was obtainable with STED microscopy, would be helpful to understand the protein distribution and composition of peroxisomal membrane proteins better, which might help to link them to a physiological function. As the density of the epitopes, recognized by antibodies against our proteins of interest, on the peroxisomal membrane is very high, verifying their colocalization is challenging. Therefore, we aimed to establish a combination of ExM and STED microscopy for the analysis of intracellular organelles.

To achieve this goal several problems needed to be addressed. First, the staining of the sample after expansion is needed to be bright enough to perform two‐colour STED imaging, as a linear expansion factor of 4 (which is in general obtained by ExM)^[2]^ results in a 4^2^‐fold increase in area and a 4^3^‐fold increase in volume. During expansion, the fluorophores at the epitope thus move away from one another, increasing the dye‐to‐dye distance and lowering their density.

Second, the isotropy of the expansion needed to be validated. In the literature, several approaches can be found to validate the isotropic expansion of the sample embedded in acrylamide gels. Often, a vector distortion field is used, which is working well in expanded tissue slices.^[2]^ Here, the sample is imaged before and after expansion and any distortions become apparent when the images are compared. However, this approach is not appropriate for the imaging of single cells, as exact same cells before and after expansion are difficult to find.

Therefore, we wanted to explore the congruence of the expansion factor (EF) between different organelles in the same cell and gel. Using confocal and STED microscopy, we determined the size of the nucleus, mitochondria, entire cells, and the peroxisomal matrix and membrane. We discovered variations in the values of EF, indicating that the expansion within a cell can vary, which therefore needs to be validated with carefully chosen controls.

## Results and Discussion

### Expansion of the nucleus

In this study we aimed to examine the application of expansion microscopy to address cell biological questions, potentially in combination with STED microscopy. Furthermore, we tried to find a method to validate the EF that does not require imaging the same cell in the unexpanded and expanded state, but that rather measures a large number of cells, that is, to obtain the EF by examining microscopic structures inside the sample instead of the macroscopic EF of the whole gel.

**Figure 1:**
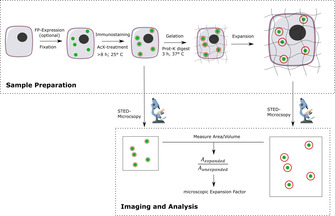
Expansion microscopy of intracellular organelles. HEK293 cells were grown on glass coverslips and transfected to achieve fluorescent protein (FP)‐expression (optional). Cells were then fixed, immunostained, treated with the crosslinking reagent AcX and embedded into the gel. Subsequently, the sample was treated with proteinase K, which digests all cellular proteins to peptides, that are crosslinked to the gel. This allows them to expand together with the gel when incubated in water. The expanded cells were imaged by conventional confocal or STED microscopy. Unexpanded controls were imaged after immunostaining. The area or volume of the structure of interest was then measured and used to calculate the expansion factor.

**Figure 2:**
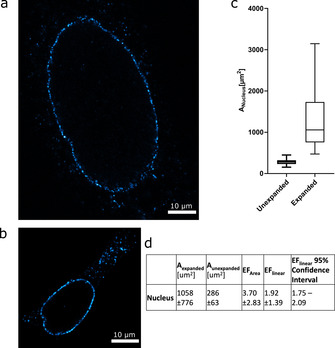
Expansion of the nucleus. a) HEK293 cell immunolabelled with an antibody against the nuclear pore complex protein NUP153 in an expanded gel. For imaging, the confocal plane with the maximal extent of the nucleus was chosen. The size of the nucleus was measured by manually tracing the NUP153 signal, and the resulting area was recorded for analysis. b) For unexpanded cells treated in the same manner, the analysis was performed analogously. c) Box‐whisker plot comparing the measured areas for maximal extent of the nucleus between expanded and unexpanded cells. The median value is denoted by the bar; the box shows the quartile ranges. Whiskers extend from the 5th to the 95th percentile. d) Expansion factor calculated from pooled median areas of unexpanded (*n*=80) and expanded (*n*=54) nuclei across two independent replicates. The EF was calculated using median values.

We chose the cellular nucleus as a first organelle to measure the EF. We thus immunostained the nuclear pore complex in fixed HEK293 cells with a primary antibody against Nucleoporin 153 (NUP153) and a dye conjugated secondary antibody. Afterwards, the sample was gelled and expanded (see Figure 1 for an overview of the procedure). Using confocal microscopy, the cells were optically sectioned and the maximal extent of the nucleus determined and imaged. In these images, the extent of the nucleus was then manually traced and its area recorded (Figure 2). By comparing the area from nuclei of expanded (Figure 2a) and unexpanded (Figure 2b) cells a microscopic EF was calculated, shown in Figure 2c and d. The linear expansion factor is calculated by taking the square root of the expansion in area or the third root of the expansion in volume. The volumes can be calculated from a series of images (*z*‐stack), since the distance between the slices is known. Figure 2d shows significant standard deviations for the measured areas, and the resulting error in the EF is also large. This is due to the inherent variance of biological systems. In the case of the nucleus, cells are morphologically different and are at different points in the cell cycle at the time of fixation. However, the narrow 95 % confidence interval shows that the EF can be reliably determined in this fashion if the sample size is sufficiently large.

The microscopic EF of 1.9 obtained from the NUP153 staining deviated from that determined from the macroscopic EF of 4.1 of the whole gel. Under the assumption of isotropic expansion, this discrepancy is rather surprising and unexpected. However, Pesce and colleagues reported similar discrepancies when examining nuclear pore complexes as potential intrinsic reporters for the expansion factor:^[9]^ In this study, the EF of the gel was reported to be 5.0, while the EF determined through the distance between nuclear pore complexes was reported as 3.8. The EF calculated from the radii of NPC before and after expansion was reported as 4.3. Another study found a linear microscopic expansion factor of nuclei which matches exactly that of the gel (4.1). This was reported for isolated barley nuclei, but only when employing a slightly modified ExM protocol using heat denaturation, which in turn resulted in impairments in chromatin structure after expansion.^[10]^ In another example, when examining rat hepatocytes, Pernal and colleagues reported an EF of 4.71 for the whole cell, whereas the nucleus only expanded by a factor of 3.86.^[11]^ The same study also examined human primary skeletal muscle cells. Crucially, it showed that the relative ratio of (heterochromatin‐containing) DAPI‐stained areas of the cell to the area of the whole nucleus changed during expansion because the heterochromatin‐containing regions expanded to a lesser degree than the nucleolus. In the same cells, the EF determined via the width of myosin fibres was shown to be 2.7. For these nuclei, the length‐to‐width‐ratio also changes during expansion. The authors furthermore list fragmentary pieces of evidence for differential expansion from several other publications employing ExM. In conjunction with our data, this points to the conclusion that the nucleus expands anisotropically and that there are further examples of differences between EFs determined by macroscopic inspection of the whole gel and EFs of cellular structures, which has to be considered for final structure‐size determination.

### Expansion of the cell area and the mitochondrial network

**Figure 3:**
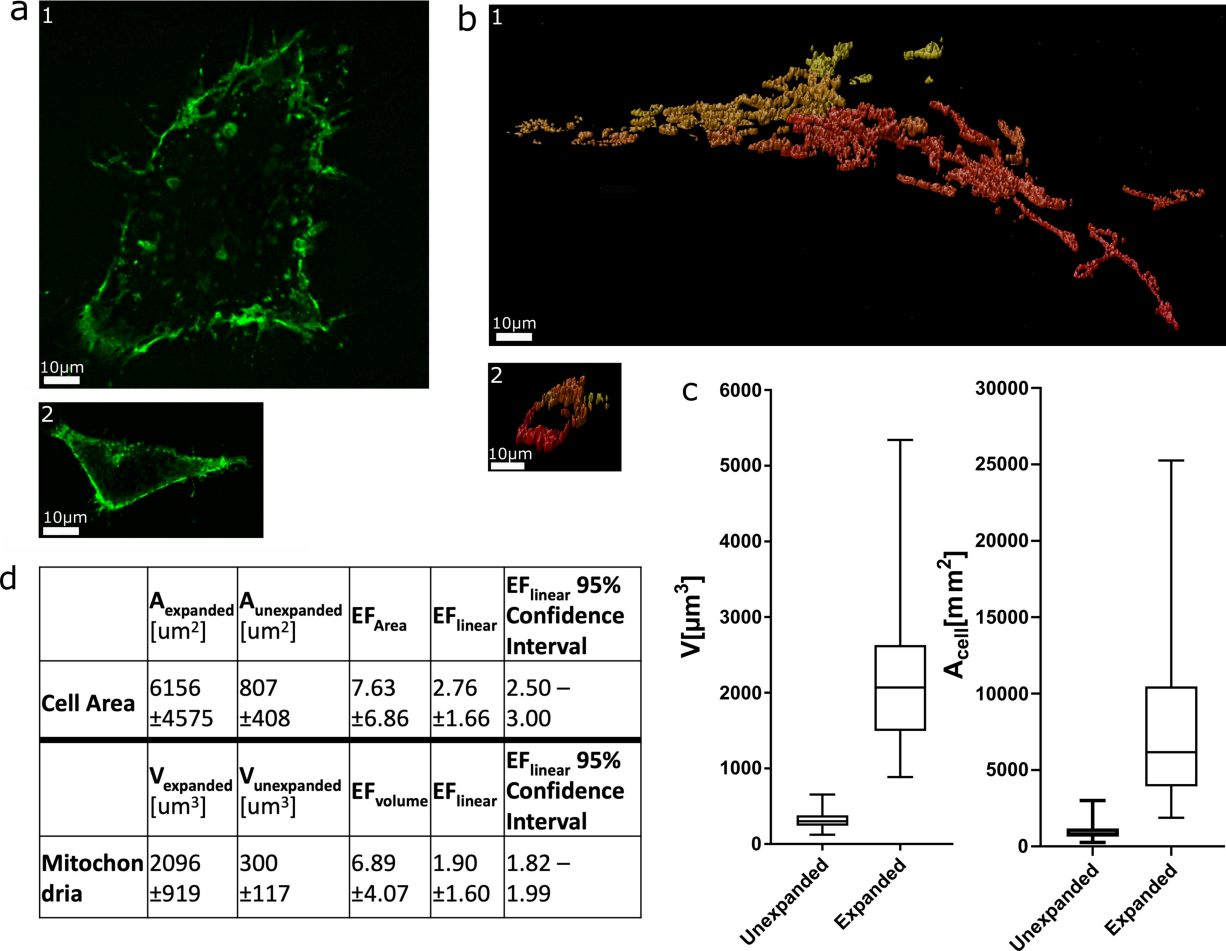
Expansion of the cell area and mitochondria within the same cells. To stain the plasma membrane, HEK293 cells expressing GPI‐GFP were additionally immunolabelled with antibodies against the mitochondrial outer‐membrane protein TOM20. The cells were expanded and imaged as *z*‐stacks on a spinning disc microscope setup. a) Example images of 1) expanded and 2) unexpanded cells expressing GPI‐GFP on the cell membrane. The *z*‐slice with the maximum extent of the cell was chosen, and the cell area was measured manually. b) Example surface renderings of immunostained mitochondria derived from the z‐stacks of 1) expanded and 2) unexpanded cells are shown. The red‐to‐yellow shading of the surface renderings illustrates the depth, where yellow objects are further away from the viewer. The *z*‐stacks were thresholded, and the volume of all voxels was summed to obtain the volume of the whole mitochondrial network. c) Box‐whisker plot showing measured volumes for TOM20 and areas for GPI‐GFP. The median value is denoted by the bar; the box shows the quartile ranges. Whiskers extend from the 5th to the 95th percentile. d) Expansion factors calculated from median volumes of the mitochondrial network of unexpanded (*n*=76) and expanded (*n*=80) cells. The expansion factor for cell areas was calculated from median areas of unexpanded (*n*=206) and expanded (*n*=72) cells. The pooled data shown were obtained from three independent replicates.

We next attempted to use the volume of the whole cell and the volume of the mitochondrial network to measure the microscopic EF. To this end, the plasma membrane was labelled by expression of glycosylphosphatidylinositol (GPI)‐anchored GFP in HEK293 cells and an ATTO488‐conjugated single‐domain antibody directed against GFP was used to boost this signal. In the same cells, mitochondria were immunostained for TOM20, a marker for the outer mitochondrial membrane. Expanded and unexpanded cells where then imaged as z‐stacks on a spinning disc confocal microscope setup. The signal intensity of the GPI‐GFP plasma membrane staining after expansion was too low to measure the volume of the cell accurately. This was most likely due to the aggressive permeabilization required for the ExM protocol, which destabilized the plasma membrane. Hence a lot of the GPI‐GFP was washed away and after expansion the remaining signal was too weak to enable the reconstruction of a 3D image of the cell that could be used to estimate its volume. Instead, the *z*‐slice with the maximum extent of the cell was chosen and its area recorded (Figure 3a). The antibody‐based labelling of the mitochondrial outer membrane using the TOM20 antibody gave a better signal‐to‐noise ratio. The TOM20 signal was segmented via thresholding in every stack and then all the voxels (dimensions per voxel: 0.135×0.135×0.370 μm^3^) were counted to obtain the volume of the organelle (Figure 3b). While comparison of the cell areas obtained a linear EF of 2.7, comparison of mitochondrial volumes between expanded and unexpanded cells yielded a linear EF of 1.9 (Figure 3c and d).

A problem for measuring the volume of mitochondria was the loss of fluorescence intensity during the expansion. For measuring the volume of the mitochondria in the reconstructed 3D images, the image was thresholded, which is not easy when the brightness of the two samples (expanded and unexpanded) varies. Although this might have led to false‐negative detection of voxels in the expanded cells, the signal was strong enough to recreate the shape of the organelles quite efficiently. This effect alone cannot explain the difference in the linear EF of mitochondria (1.9) to the one of the gel (4.1), indicating again that the expansion of intracellular structures was much less than that of the gel.

### The peroxisomal membrane expands more than the peroxisomal matrix

As the initial motivation for the study was to elucidate the protein distribution of organelle membranes, like the peroxisomal membrane, with a combination of ExM and STED, we wanted to measure the expansion of this organelle. Therefore, the matrix and membrane of peroxisomes were labelled and imaged with STED microscopy before and after expansion.

In order to obtain good signal‐to‐noise ratios when using ExM, the most common technique is the expression of the protein of interest fused to a fluorescent protein. Then polyclonal, fluorophore‐conjugated antibodies are applied that are directed against the fluorescent protein. This approach gives a strong signal and is feasible for staining of whole organelles with specific markers such as GFP containing a peroxisomal targeting signal type 1 (PTS1) for the peroxisomal matrix. However, this approach becomes infeasible when colocalization of small, motile proteins and their interaction partners are to be examined. The bulky, fluorescent protein may change the localisation of the protein of interest or may block its binding domain for an interaction partner. Hence the only option here is immunostaining with a primary antibody against the protein of interest.

**Figure 4:**
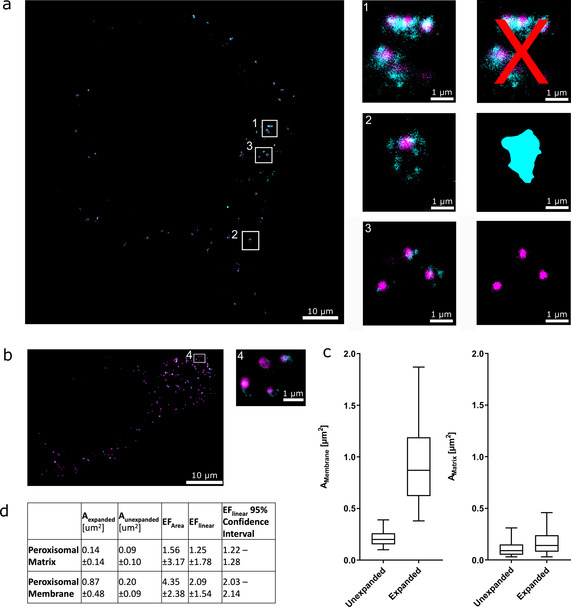
Expansion of peroxisomes. HEK293 cells expressing GFP‐PTS1 were immunolabelled with an antibody against PEX14 (cyan), expanded and imaged in two‐colour STED. The GFP signal was boosted with an ATTO488‐labelled nanobody against GFP (magenta). a) One expanded HEK cell; insets are highlighted on the right with a visualization of the data analysis (right). a1) As clear assignment of the PEX14 signal in clustered peroxisomes was not possible, these were excluded from the analysis. a2) In more isolated peroxisomes, the peroxisomal membrane was manually traced according to the PEX14 signal, and the area was determined for analysis. a3) For the peroxisomal matrix, the GFP‐PTS1 signal was thresholded, and its area was determined automatically. b) An unexpanded cell treated and stained by the same method was used for the gels. Areas were measured analogously to the analysis of expanded cells. c) Box‐whisker plots showing areas of peroxisomal matrix and membrane before and after expansion. The median value is denoted by the bar; the box shows the quartile ranges. Whiskers extend from the 5th to the 95th percentile. d) Median areas of unexpanded (*n*=744) and expanded (*n*=654) peroxisomal membranes were used to calculate the expansion factor. Similarly, for the peroxisomal matrix, median areas of unexpanded (*n*=3657) and expanded (*n*=3322) matrices were compared. The pooled data shown were obtained from three independent replicates.

We thus labelled the peroxisomal matrix with GFP‐PTS1. The peroxisomal targeting signal directed the GFP across the peroxisomal membrane into the lumen of the peroxisomes. Then, an ATTO488‐conjugated‐anti‐GFP single domain antibody was applied to enhance (or boost) this signal. To label the membrane, a polyclonal antibody against PEX14 was used. This antibody has a very high affinity to its target and gives a good signal to noise ratio. When used in combination with stable dyes compatible with STED microscopy, such as Aberrior STAR RED, the labelling density is sufficient for ExM‐STED. A representative image of the obtained two‐colour STED images of peroxisomal matrix and membrane is shown in Figure 4a) shows expanded cells in the gel, while b) shows unexpanded cells. We have to note that HEK cells, when mildly permeabilized, conventionally embedded, and imaged using STED microscopy allow resolution of greater detail^[8]^ than that achieved in the unexpanded cells shown here. This effect is most likely caused by the harsh permeabilization needed for the gelation.

The size of the peroxisomal membrane could not be measured automatically, as the signal for PEX14 appeared to be dotted along the membrane. Assigning the PEX14 signal was not possible for clustered peroxisomes (Figure 4a‐1), as it could not be distinguished to which peroxisome the signal belongs. These peroxisomes were thus excluded from further analysis. For peroxisomes whose borders could be defined by the membrane staining (Figure 4a‐2), the PEX14 signal was manually traced to obtain the outline of the peroxisomal membrane and then its extent was recorded. For the peroxisomal matrix, we thresholded the boosted GFP‐PTS1 signal and then measured the area of the matrix using the Fiji‐Plugin “Particle Analyzer”^[12]^ (Inset 3 of Figure 4a). As before, we thus obtained two sets of areas for expanded and unexpanded cells that were employed to calculate the linear EF. With a median expansion factor of 4.1 for the gel, the linear microscopic EF for the peroxisomal membrane was 2.1, while the expansion of the peroxisome matrix was considerably smaller. The linear EF for the peroxisomal matrix was 1.3. This result can however not be attributed to a poor signal‐to‐noise‐ratio, since the chosen labelling approach also gave strong fluorescence in the expanded gel. Instead, this result is most likely caused by the dense environment of the peroxisomal matrix, which in some cases even contains a crystalloid protein core.^[13]^ It has also been shown that peroxisomes do not expand like other organelles, including ER, endosomes, lysosomes or mitochondria, upon incubation of cells in hypotonic solution.^[14]^ This is probably also due to the dense protein matrix in the lumen of these organelles. During the digestion step, proteinase K probably cannot cleave proteins in this environment efficiently, resulting in incomplete expansion of these protein structures afterwards. To overcome this issue, we tried to prolong the digestion with proteinase K, but this led to significant loss of signal (data not shown. We also tried to use a monoclonal antibody against PEX5, the peroxisomal import receptor, which is shuttling between the cytosol and the peroxisomal membrane to import PTS1‐containing cargo proteins. At the membrane, PEX5 is interacting with PEX14, triggering the translocation of the cargo protein.[^15^, ^16^, ^17^] The monoclonal PEX5 antibody used here, only binds to PEX5 when it is located at the peroxisomal membrane and not to the cytosolic pool of the shuttling receptor.^[18]^ Here, we obtained good STED images using PEX14 and PEX5 in a dual colour STED colocalization study^[8]^ but the signal with the PEX5 antibody was too weak for ExM‐STED (data not shown). The weak signal might be due to the fact that monoclonal antibodies bind to only one epitope on their target protein, therefore the use of polyclonals is recommended for ExM‐STED, as these can bind multiple epitopes on their target protein.

### Organelles can expand differently within one cell

Our data clearly indicates that the EFs of different organelles, and in case of peroxisomes even distinct regions of the same organelle, differs significantly. Although the linear expansion factor of the gel was consistently 4.1, the EF of the measured cellular compartments ranged from 2.7 (cell area) to 1.3 (peroxisomal matrix; Table 1). Indeed, differences in EF were also encountered when applying ExM to bacteria, due to biochemical heterogeneity of their cell walls. In some cases, it was possible to abolish these differences in EF by digesting the cell wall prior to expansion.^[19]^ Taken together these findings clearly indicate that the expansion of subcellular structures is not isotropic within the gel and that this needs to be considered when interpreting data from single cell measurements in ExM.

**Table 1:** Expansion factors of different organelles calculated with median areas or volume and the resulting linear expansion factor.

Organelle	Expansion factor			
	Area	Volume	Linear	Linear (95 % confidence interval)
nucleus	3.70±2.83	–	1.92±1.39	1.75–1.90
mitochondria	–	6.89±4.07	1.90±1.60	1.82–1.99
cell area	7.63±6.86	–	2.76 ±1.66	2.50–3.00
peroxisomal matrix	1.56±3.17	–	1.25±1.78	1.22–1.28
peroxisomal membrane	4.35±2.38	–	2.09±1.54	2.03–2.14

### Direct comparison of individual cells before and after expansion

We also considered to compare the expansion of one organelle in the same individual cell before and after expansion, as this would be in analogy to the vector‐field‐distortion analysis that has classically been used to validate isotropic expansion in ExM (e. g., for microtubule).^[4]^ By training to find the same cell before and after expansion we faced a number of hindrances that made it impossible to realize this in a reliable fashion. When examining a coverslip with HEK cells and the resulting gel, it was difficult to clearly identify the same cell in samples pre‐ and post‐ expansion, because most cells were morphologically quite similar. An identification was in addition complicated due to the large difference in fluorescence brightness before and after expansion We thus analysed many cells within large sample sizes independent of pre‐ and post‐expansion instead, to avoid erroneous assignment of single cells.

### Consideration of other organelles

We also considered investigating the expansion factor of further organelles. For example, microtubules have been used before to validate isotropic expansion.[^4^, ^6^] However, to in detail explore and compare values of the expansion factor, we found it more reliable to employ areas instead of one‐dimensional structures such as microtubules.

Labelling of the mitochondrial matrix could also be considered to compare the expansion factor of the mitochondrial membrane to that of the matrix. However, for our expansion experiments (especially when combined with STED) there is, as also outlined below, a further need for very bright staining, since expansion leads to a “dilution” of signal. For example, even for a usually efficient stain, such as of TOM20 in the mitochondrial membrane, we observed such a pronounced lowering of signal after expansion, that the signal‐to‐noise ratio of the resulting images was rather low and we were only able to evaluate the data after deconvolution (and thus denoising). Any staining of the matrix would be dispersed throughout the whole mitochondrion and thus be less concentrated after expansion, compared to for example the high concentration of the actively imported peroxisomal membrane marker.

Nevertheless, we believe that the matrix of mitochondria will probably expand more or less equally to the membrane, as it does in hypotonic solution.^[14]^ On a note, compared to mitochondria, the crystalloid core of peroxisomes is a very organelle‐specific property and therefore the difference of the expansion of peroxisomal membrane and matrix an extreme example. Nevertheless, this highlights that the differences in expansion can be dramatic and need to be considered in any of such experiments. We anticipate to use expansion microscopy as a tool to study the properties of the crystalloid core of peroxisomes in more detail.

### Alternative approaches to increasing the fluorescence intensity

The loss of fluorescence intensity encountered in ExM‐STED, due to a combination of loss of fluorophores and a physical “dilution” of the signal during expansion of the sample, poses a major challenge. Several approaches have been used to increase the labelling density in the pre‐expansion sample in order to attain a sufficient labelling density in the expanded gel. One option is to first use primary antibodies conjugated with biotin on the unexpanded cells, followed by treatment with fluorophore‐conjugated secondary antibodies directed against these primary antibodies. The expanded gel is then perfused with streptavidin conjugated fluorophores. This results in an increased labelling density when compared to conventional immunostaining.^[6]^ A variant of this method allows signal amplification through iterative treatment of the sample with biotin‐ and dye‐conjugated secondary antibodies and dye‐conjugated streptavidin. While this method gives a fluorophore density high enough to reach the maximum STED resolution in an expanded sample, it also introduces a localisation error with each amplification cycle, due to the resulting distance between the protein of interest and the fluorophore.^[20]^ An alternative to expressing the protein of interest conjugated with a fluorescent protein is to attach the AviTag peptide sequence, consisting of only 16 amino acids. The small peptide is less likely to interfere with the proteins motility and to block binding domains than the bulky structures of a fluorescent protein. This previously demonstrated method^[20]^ then uses the BirA biotin ligase to biotinylate the peptide. The sample can then be treated with the same iterative treatment as outlined above.

The heterogeneous loss of fluorescence comes with another challenge for application in cell biology, as discussed by Pesce and colleagues.^[9]^ They analysed NUP153, which forms a symmetrical octagon in the nuclear pore complex, and noticed heterogeneous loss of signal due to polymerization and digestion in the ExM protocol. For well‐explored epitopes like the nuclear pore, other data (mainly from CryoEM) can be consulted to compare the image obtained from an expanded cell. However, for proteins of interest where little prior knowledge exists and where their (co)localization or structure is to be examined, noticing artefacts due to heterogeneous signal loss becomes more challenging.

## Conclusion

Expansion microscopy is a sophisticated tool for the analysis of tissue slices, as the structures can be easily compared between expanded and unexpanded samples. Here a vector‐distortion field can be applied to visualize and detect anisotropic expansion due to biomechanical heterogeneity of the sample. Furthermore, different parts of tissue slices often have varying refractive indices. These lead to aberrations that limit the depth to which a slice of tissue can be imaged. This problem is partly overcome by ExM as the gel has almost the same refractive index as water, which alleviates this issue. Therefore, ExM improves deep‐tissue imaging.

Our data points to the conclusion that it might be difficult to define one epitope or structure inside cells as a reference and then generalize isotropy and EF from it. The biochemical heterogeneity of cellular organelles makes it necessary to validate the expansion on exactly the epitope that is to be measured. As the expansion factors we measured differ for all analysed organelles, and in case of peroxisomes even for structures within the same organelle, it becomes clear that ExM on subcellular structures is quite challenging. Peroxisomes are a difficult organelle for ExM as they have a protein‐rich crystalline core, which is difficult to expand because of its special biochemical properties. This might explain the very low EF for the peroxisomal matrix, but it is also clear that the big variety of EFs measured with different organelles is a problem for imaging intracellular details with ExM.

## Experimental Section

**Cell culture**: HEK293 cells (ATCC, Manassas, VA, USA) were maintained in a culture medium consisting of DMEM with 4500 mg/L glucose, 110 mg/L sodium pyruvate supplemented with 10 % fetal calf serum, glutamine (2 mM) and penicillin‐streptomycin (1 %). The cells were cultured at 37 °C/8.5 % CO_2_. Cells were grown on #1.5 cover slides of 18 mm diameter. For the analysis of the peroxisomal matrix, the cells were transfected with 0.5 μg GFP‐PTS1 [21] per dish using Lipofectamine 2000 transfection reagent (Invitrogen). Transfected cells were fixed for immunostaining 24 h after transfection.

**Immunostaining**: Cells were fixed with 3 % Formaldehyde for 20 min and permeabilized with 0.1 % Triton X‐100 for 10 min. Samples were then blocked (2 % BSA, 5 % FCS in PBS, for 1 h at RT) and incubated in the primary antibody in dilutions shown in table 2. Subsequently, samples were incubated with the secondary antibody (Table 3) and treated with 0.1 mg/ml 6‐((acryloyl)amino)hexanoic acid, succinimidyl ester (AcX) (ThermoFisher) for at least 12 h.

**Table 2:** Primary antibodies.

Epitope	Species	Clonality	Dilution	Source
aPEX14	rabbit	polyclonal	1 : 400	see ref. [19]
NUP153	mouse	monoclonal	1 : 400	Abcam, QE5
TOM20	rabbit	polyclonal	1 : 200	Santa Cruz Biotechnology, FL145

**Table 3:** Secondary antibodies.

Dye	Reactivity	Species	Dilution	Source
Aberrior STAR Red	anti‐rabbit	goat	1 : 250	Aberrior 2‐0012‐011‐9
Aberrior STAR Red	anti‐mouse	goat	1 : 250	Aberrior 2‐0002‐011‐2
ATTO488	recombinant, monoclonal anti‐GFP single‐domain antibody (sdAb) fragment	alpaca	1 : 1000	Chromotek

**Gelation, digest and expansion**: Gelation was performed as previously described [2]. Briefly, monomer solution (1x PBS, 2 M NaCl, 8.625 % sodium acrylate, 2.5 % acrylamide, 0.15 % *N*,*N′*‐methylenebisacrylamide) was mixed, frozen in aliquots, and thawed before use. Monomer solution was used at 4 °C. Concentrated stocks of ammonium persulfate (APS) and tetramethylethylenediamine (TEMED) were added to the monomer solution up to 0.2 % each. Polymerisation was allowed to proceed for 15 min at RT and then 1 h at 37 °C to allow complete gelation. The gel was then cut asymmetrically, and the length of its longest side was recorded. Gels were then removed from the gelation chamber and fully immersed in digestion buffer (50 mM Tris (pH 8), 1 mM EDTA, 0.5 % Triton X‐100, 1 M NaCl) with 8 U/mL of Proteinase K (BioVision) added immediately before use. Digestion was performed for 3 h at 37 °C. For expansion, gels were placed in doubly deionized water (ddH_2_O) for 4 h. ddH_2_O was replaced every 30 min until the maximum expansion of the gel was reached. The length of the longest side was recorded again and used to calculate the EF of the gel. This consistently gave an EF ranging between 3.9–4.2 with a median EF of 4.1.

**Mounting**: For mounting of gel‐embedded cells, FluoroDish glass bottom dishes (World Precision Instruments) were coated with 0.1 mg/ml Poly‐l‐lysine for 1 h at 37 °C and then washed once with ddH_2_O. Expanded gels were placed on the glass and all excess water removed. After allowing adhesion for 5 min, the dish was placed in the sample holder of the microscope. The top of the gel was covered with a small amount of ddH_2_O to prevent evaporation.

For unexpanded controls the cells were grown on glass slides, then fixed, stained and incubated in AcX and subsequently imaged. For imaging the coverslip was mounted in an imaging chamber and covered with PBS.

**Image acquisition**: Images of GFP‐PTS1 expressing HEK293 cells immunostained for either NUP153 or PEX14 were acquired on a Leica SP8 3× STED Microscope with a HC PL APO 86× 1.2 NA objective with motorized collar correction. The white‐light laser was used for excitation at 488 and 640 nm with depletion via the 592 and 775 nm STED laser, respectively. NUP153 signal was acquired in confocal mode only. When using two depletion lasers of different wavelengths, only one STED‐channel can be acquired at a time. Hence, for GFP‐PTS1/PEX14 stained cells, acquisition was performed frame‐by‐frame. First, PEX14 signal was acquired in STED with parallel acquisition of GFP‐PTS1 signal in confocal. Then, GFP‐PTS1 signal was acquired in STED. To correct for any drift in between acquisition of the frames, the Fiji‐Plugin “Correct 3D Drift” was used to align the channels according to the GFP‐PTS1 signal.

GPI‐GFP expressing HEK293 cells immunostained for TOM20 were imaged on a Zeiss Cell Observer SD with a Yokogawa CSU‐X1 M 500 Dual Cam spinning disc, Hamamatsu Orca Flash 4.0 V2 sCMOS camera and a LD C‐Apochromat 40× 1.1 NA objective. Excitation of ATTO488‐nanobody boosted GFP signal was done at 488 nm, while Aberrior STAR RED was excited at 635 nm.

**Image processing and data analysis**: TOM20 images acquired on the spinning disc setup were deconvolved with Huygens (Scientific Volume Imaging), using classical maximum likelihood estimation (CMLE). All image analysis was performed using Fiji (ImageJ). In particular, the “Particle Analyzer” and “Voxel Counter” scripts were used to obtain areas and volumes respectively.^[12]^ Graphing and statistical analysis was performed using GraphPad. In all box‐whisker plots, the median value is denoted by the bar and the box shows the quartile ranges. Whiskers extend from the 5th to the 95th percentile. The EF in all tables was calculated using median values.

**Antibodies**: Primary and secondary antibodies are listed in Tables 2 and 3, respectively.

## Conflict of interest

The authors declare no conflict of interest.
